# Range-Expanding Populations of a Globally Introduced Weed Experience Negative Plant-Soil Feedbacks

**DOI:** 10.1371/journal.pone.0020117

**Published:** 2011-05-23

**Authors:** Krikor Andonian, José L. Hierro, Liana Khetsuriani, Pablo Becerra, Grigor Janoyan, Diego Villarreal, Lohengrin Cavieres, Laurel R. Fox, Ragan M. Callaway

**Affiliations:** 1 Department of Ecology and Evolutionary Biology, University of California Santa Cruz, Santa Cruz, California, United States of America; 2 Facultad de Ciencias Exactas y Naturales, Universidad Nacional de La Pampa, Santa Rosa, Argentina; 3 CONICET, La Pampa, Santa Rosa, Argentina; 4 Institute of Botany of the Georgian Academy of Sciences, T'bilisi, Georgia; 5 Departamento de Ecosistemas y Medio Ambiente, Facultad de Agronomía e Ingeniería Forestal, Universidad Católica de Chile, Santiago, Chile; 6 Acopian Center for the Environment, American University of Armenia, Yerevan, Armenia; 7 Departamento de Botánica, Universidad de Concepción, Concepción, Chile; 8 Division of Biological Sciences, University of Montana, Missoula, Montana, United States of America; Duke University, United States of America

## Abstract

**Background:**

Biological invasions are fundamentally biogeographic processes that occur over large spatial scales. Interactions with soil microbes can have strong impacts on plant invasions, but how these interactions vary among areas where introduced species are highly invasive vs. naturalized is still unknown. In this study, we examined biogeographic variation in plant-soil microbe interactions of a globally invasive weed, *Centaurea solstitialis* (yellow starthistle). We addressed the following questions (1) Is *Centaurea* released from natural enemy pressure from soil microbes in introduced regions? and (2) Is variation in plant-soil feedbacks associated with variation in *Centaurea*'s invasive success?

**Methodology/Principal Findings:**

We conducted greenhouse experiments using soils and seeds collected from native Eurasian populations and introduced populations spanning North and South America where *Centaurea* is highly invasive and noninvasive. Soil microbes had pervasive negative effects in all regions, although the magnitude of their effect varied among regions. These patterns were not unequivocally congruent with the enemy release hypothesis. Surprisingly, we also found that *Centaurea* generated strong negative feedbacks in regions where it is the most invasive, while it generated neutral plant-soil feedbacks where it is noninvasive.

**Conclusions/Significance:**

Recent studies have found reduced below-ground enemy attack and more positive plant-soil feedbacks in range-expanding plant populations, but we found increased negative effects of soil microbes in range-expanding *Centaurea* populations. While such negative feedbacks may limit the long-term persistence of invasive plants, such feedbacks may also contribute to the success of invasions, either by having disproportionately negative impacts on competing species, or by yielding relatively better growth in uncolonized areas that would encourage lateral spread. Enemy release from soil-borne pathogens is not sufficient to explain the success of this weed in such different regions. The biogeographic variation in soil-microbe effects indicates that different mechanisms may operate on this species in different regions, thus establishing geographic mosaics of species interactions that contribute to variation in invasion success.

## Introduction

Species invasions pose a serious threat to biodiversity, cause massive economic losses, and are at the forefront of some of the most interesting conceptual topics in ecology and evolutionary biology [1], [2]. While biological invasions have received much recent attention from ecologists [3]–[5] most research has been directed at identifying locally occurring mechanisms that drive invasions [6], [7]. However, invasions are primarily biogeographical phenomena that may occur over broad spatial scales [4]. Thus, examining the mechanisms that drive invasions over broad spatial scales and between native and non-native ranges will increase our understanding of exotic invasions.

Introduced species vary in dispersal, colonization, and impact within and among the different regions into which they have been introduced [8], [9]. This variability may reflect differences in abiotic environments or species interactions among regions and shed light on the mechanisms responsible for invasion [4]. In their non-native ranges, invasive species interact with novel suites of natural enemies, mutualists, and competitors that can affect their successful establishment and spread [10], [11]. One of the main hypotheses for successful plant invasions is the Enemy Release Hypothesis (ERH), which suggests invasive species leave behind their native natural enemies [12], [13]. Alternatively, invasive species may encounter mutualists in introduced regions that are more beneficial than mutualists in native regions, a pattern observed in plant-soil microbe interactions [14]. However, these mechanisms are not mutually exclusive, and invasion success may ultimately be due to a mosaic of factors operating in different regions [15]. Thus, introduced species' newly acquired natural enemies, mutualists, and competitors can often determine whether they become simply naturalized, or achieve the high density and ecological impact characteristic of invasive species [10], [11].

The microbial pathogens and mutualists that plants encounter below ground can have strong impacts on the assembly of plant communities [16], [17] and exotic plant invasions [18]–[20]. The combined effects of pathogens and mutualists are often studied in the context of “plant-soil microbe feedbacks (PSFs),” where plant roots accumulate unique, species-specific assemblages of microbes that can have either positive or negative effects on their hosts or heterospecifics [21]. Negative feedbacks enhance coexistence and plant diversity through negative frequency dependence, while positive feedbacks can result in low-diversity communities dominated by few species [21], [22]. Importantly, negative feedbacks are generally stronger for plants and soil microbes in their native ranges than for invaders and soil microbes in non-native ranges [19], [22]. Although cross-continental comparisons of plant-soil feedbacks have demonstrated that soil biota can have powerful effects on invasions [20], [22]–[24], we know little about variation among different invaded ranges.

*Centaurea solstitialis* (yellow starthistle, Asteraceae; hereafter referred to as *Centaurea*) is an annual forb native to Eurasia that has been introduced throughout the world. In its native range, it occurs in isolated populations at low densities, commonly at ∼5 plants/m^2^ (K. Andonian, *unpublished data*). In some introduced regions such as Argentina and California, *Centaurea* is highly invasive, occupies large and dense patches often exceeding 200 plants/m^2^, and is spreading rapidly [25], [26]. However, in other regions where it has been introduced such as Chile, *Centaurea* spreads slowly, does not have strong impacts, occupies small patch sizes, and is commonly found at low densities averaging ∼20 plants/m^2^ (L. Cavieres, *unpublished data*). This biogeographical variation in the abundance and apparent impact of *Centaurea* suggests variation in the importance of the mechanisms that drive its success.

*Centaurea* may have been released from natural enemy pressure in introduced regions, but preliminary findings (K. Andonian, *unpublished data*) show that *Centaurea* actually experiences *more* insect attack in its introduced range in California than it does in native populations in Eurasia [27]. *Centaurea* can also alter soil microbial communities in ways that may enhance its own competitiveness [28], [29]; however, we still do not know how soil microbes influence populations throughout the broad global distribution of this invader. Our goal in this study was to understand the influence of soil microbes throughout the native and introduced regions of *Centaurea*. Specifically, we conducted greenhouse experiments using seeds and soils from four regions to address the following questions: (1) Is *Centaurea* released from natural enemy pressure from soil microbes in introduced regions? and (2) Is variation in plant-soil feedbacks correlated with variation in *Centaurea*'s invasive success across native and introduced regions?

By sampling populations from regions where *Centaurea* is native, spreading, *and* naturalized, we have obtained a geographically broad, robust snapshot of the ecological variation of its interactions with soil microbes and how they may contribute to its spread.

## Methods

### Study System and Biogeographical Regions

*Centaurea solstitialis* is native to the eastern Mediterranean and the Caucasus region in Eurasia, but now grows on every continent except Antarctica [30], [31]. Because *Centaurea* has been introduced to many regions with variable success, we sampled populations from three regions in which *Centaurea* has been introduced (Argentina, Chile, and California), and from populations in its native region in Eurasia, focusing on populations in the Republics of Armenia and Georgia. We categorized Argentina and Chile as two separate regions because they are separated by a major biogeographic barrier, the Andes, and thus have very different climates and plant communities. Chile has a Mediterranean climate characterized by summer droughts with plant communities dominated by annual grasses, much like California, while Argentina receives summer rains with plant communities dominated by perennial grasses. However, Eurasian populations from the Republics of Georgia and Armenia both lie within the Caucasus Mountains, with similar climate and plant communities, and thus represent a single ecological region in this study. According to current estimates of introduction history, *Centaurea* is believed to have first been introduced to the Americas in Chile, from Chile into California in ∼1850 [32], and then into Argentina in ∼1870 [33].

### Soil and Seed Collections

We collected soil samples from six *Centaurea* populations per region in an effort to sample a broad range of soil microbes interacting with *Centaurea*, for a total of 24 populations (Table S1). These populations were chosen if *Centaurea* occurred at densities within one standard deviation of the mean densities for populations in each region and were at least 10 km apart. From each population, we collected 4 L of soil from the top 15 cm using a shovel sterilized in bleach (6% NaOCl solution). All soils were collected during the summer when *Centaurea* was at peak biomass and allowed to slowly air dry to mimic natural drought conditions.

We used seeds collected from one *Centaurea* population per region that was not included in the soil collections (Table S1) to avoid potential local bias that may confound comparisons. In addition to *Centaurea* seeds, we collected seeds from one population of each of three locally occurring grass species in each region (Table 1) for ‘soil training’ treatments (see below). We chose grass species that were locally abundant and in many cases were not native to the region.

**Table 1:** Grass species used to train soils during the first phase of the plant-soil feedback experiment.

Argentina	California	Chile	Eurasia
*Nassella tennuis*	Bromus diandrus*	Bromus diandrus*	*Bromus squarrosus*
*Piptochaetium napostaense*	Avena fatua*	Avena barbata*	*Hordeum lepinorum*
*Poa ligularis*	Vulpia myuros*	Vulpia bromoides*	*Poa pratensis*

All species were collected from areas in their respective regions where they are locally abundant. Asterisks indicate non-native species in their respective regions.

### Plant-Soil Feedback experiment

We used a plant-soil feedback experiment [21] to assess *Centaurea*'s interactions with soil biota within each region. Therefore, we used only sympatric seed-soil combinations in this study and compared the net effect of soil microbes on plants within regions. We grew plants in a secure rooftop greenhouse at the University of California, Santa Cruz, using 600-mL ‘conetainer’ pots (Stuewe & Sons, Inc). To eliminate potentially confounding differences in soil nutrients or physical properties, we inoculated plants with 150 mL of field soil per pot that was diluted by 20-grit blasting grade sand in a 20∶80 soil:sand mixture, then fertilized plants every 2 weeks with 1/8 strength Hoagland's solution (PhytoTechnology Laboratories™). To reduce the probability of cross contamination by soil microbes during watering we topped off all pots with a 1 cm layer of 30-grit sand.

We ‘trained’ soils from each region by growing either *Centaurea* or a combination of three grass species (Table 1) in them for 100 days. All soils were trained using seeds collected from their respective regions to maintain sympatric seed-soil combinations. After the initial training period, we autoclaved half of the soils on three successive days to sterilize soil and kill microbes. In the next stage, we planted 4 seeds from one locally occurring *Centaurea* population not used for soil collections into all pots and thinned to one individual upon germination. In total, treatments were: 4 soil regions * 6 soil populations per region * 2 soil training treatments * 2 sterilization treatments  = 96 treatments * 6–7 replicates per treatment  = 580 plants.

We monitored germination time and harvested plants 110 days after germination, separating above- and below-ground tissues. All plants were dried for 72 hours at 60°C and weighed. We used biomass as our focal response variable because *Centaurea* biomass is strongly correlated with its flower production, and thus fitness, in microcosm experiments [34].

### Common Garden Experiment

To obtain baseline differences in plant biomass of seeds used in the feedback experiment, we conducted a common garden experiment, growing *Centaurea* seeds from all four regions in identical soil environments using the same rooftop greenhouse and growing conditions as in the Feedback Experiment in a 20∶80 soil:sand mixture of potting soil and 20 grit sand. We grew 10 plants per region for 110 days after germination; all plants were harvested and measured as in the feedback experiment.

### Field Surveys

To test for correlations between results from our greenhouse studies and plant performance in the field, we collected demographic data from 3–17 *Centaurea* populations from each region that were within two standard deviations of the mean density for that region. We estimated field densities by counting total number of plants in 5 randomly placed 1 m^2^ quadrats per population, and measured patch size from 12–16 populations per region by scoring population extent as: (1) less than 30 m, (2) 30–100 m, or (3) greater than 100 m on its longest side.

### Statistical Analysis

We tested the effects of the soil treatments (region, population nested within region, training, sterilization, and all possible interactions) on germination time, root biomass, shoot biomass, total biomass, and root:shoot ratio (hereafter referred to as RSR) with a mixed model analysis of variance (ANOVA) with population nested within region as a random effect. All response variables were log transformed to meet ANOVA assumptions of normality and homoscedasticity. Specific contrasts were made using Tukey's honestly significant difference (HSD) post-hoc analyses, with **α** = 0.05.

We also calculated the effect of soil microbes using log response ratios with the following equation:
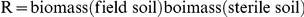
where R_m_ represents plant response to microbes, biomass (field soil)  =  mean biomass of plants grown in unsterilized field soil, and biomass (sterile soil)  =  mean biomass of plants grown in sterilized soil. Log response ratios behave as normally distributed variables and are often used to quantify the proportionate change due to experimental manipulations [35]. We calculated R_m_ for each population and then averaged R_m_ across the six populations per region to obtain the mean and variance of R_m_ for each region. The log response ratio is zero when there is no difference between the means of field and sterile soils. The response ratio is positive when microbes have positive effects that are removed by sterilization. In contrast, R_m_ is negative when microbes have negative effects so that plant performance improves in sterilized soil.

We calculated the effects of plant-soil feedbacks using the following equation:
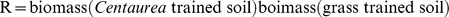
where R_fb_ represents plant response to soil training, biomass (*Centaurea* trained soil)  =  mean biomass of plants grown in soil trained by conspecific *Centaurea solstitialis* from their respective regions, and biomass (grass trained soil)  =  biomass of plants grown in soils trained by three locally occurring grass species from their respective regions. R_fb_ is positive when plants generate positive feedbacks by performing better in soils trained by conspecifics than in soils trained by grasses. Conversely, R_fb_ is negative when plants generate negative feedbacks by performing worse in soils trained by conspecifics than in soils trained by grasses. We also conducted a similar analysis examining the effects of soil training on germination time.

To determine if plant-soil feedbacks in the greenhouse are related to plant performance in the field, we plotted R_fb_ against mean field density and mean patch size class of *Centaurea* from each region.

We used Systat 12 for the specific contrasts and JMP 7.0 for all other statistical analyses.

## Results

### Plant-Soil Feedback Experiment

#### Germination time

**Figure 1:**
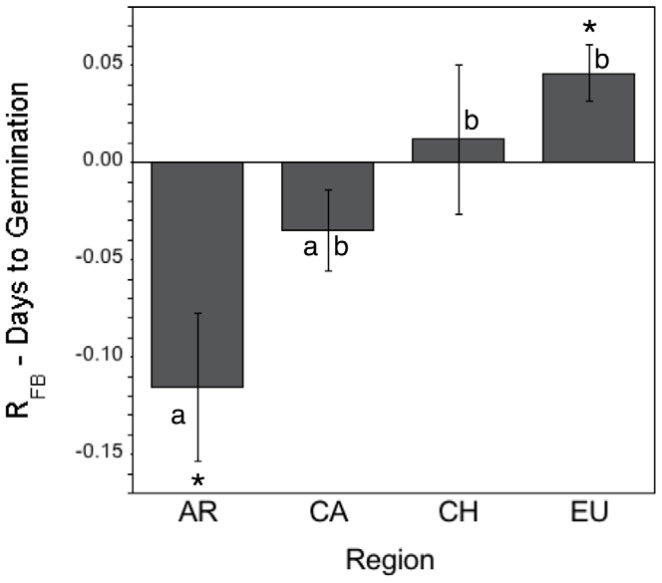
Log response ratios illustrating the net effects of soil training (e.g. plant-soil feedbacks) on germination time of *Centaurea* in soils from native and introduced regions. Negative bars represent delays in germination time in response to soil trained by conspecifics than by grasses, while positive bars represent shorter germination time in soil trained by conspecifics than by grasses. Region abbreviations are as in Table 2. Bars represent means ±1. S.E.M. Asterisks represent significant differences in germination time when grown in soils trained by *Centaurea* vs. grasses after t-test analysis. Different letters represent significant differences among regions after post-hoc Tukey HSD tests.

*Centaurea* seeds from Argentina germinated 2 days later than seeds from plants from all other regions, driving a significant effect of soil region on germination time (Table 2; Tukey HSD at **α** = 0.05), consistent with previous studies examining *Centaurea* germination [52]. Neither soil sterilization nor soil training affected germination time, but there was a marginally significant region*soil training interaction (*F*_3,20_ = 2.59, *p* = 0.08); *Centaurea* grown in Argentinean soils trained by conspecifics germinated later than in soils trained by grasses, while *Centaurea* grown in Eurasian soils trained by conspecifics germinated earlier than in soils trained by grasses (Figure 1).

**Table 2:** Means and standard errors of the main effects in the plant-soil feedback experiment.

	Region					Soil Sterilization			Soil Training		
	AR	CA	CH	EU		Field	Sterile		*Centaurea*	Grasses	
N	142	165	142	142		298	293		294	297	
Germination (days)	7.788^A^	5.437^B^	5.730^B^	5.136^B^	****	5.931	5.916		6.130	5.720	
.±S.E.M	0.674	0.145	0.168	0.136		0.237	0.222		0.285	0.157	
Root Biomass (g)	0.347^A^	0.299^B^	0.306^AB^	0.333^AB^	**	0.229	0.411	****	0.294	0.345	****
.±S.E.M	0.014	0.009	0.014	0.016		0.005	0.010		0.009	0.010	
Shoot Biomass (g)	0.180^A^	0.197^B^	0.150^C^	0.204^B^	****	0.127	0.240	****	0.174	0.192	**
.±S.E.M	0.006	0.007	0.006	0.007		0.003	0.004		0.005	0.005	
Total Biomass (g)	0.526^A^	0.497^A^	0.456^B^	0.537^A^	****	0.356^A^	0.652^B^	****	0.468	0.538	****
.±S.E.M	0.020	0.015	0.019	0.022		0.007	0.012		0.013	0.013	
Root:Shoot	1.937^B^	1.615^C^	2.102^A^	1.651^C^	****	1.861	1.772	*	1.748	1.886	***
.±S.E.M	0.048	0.042	0.058	0.052		0.034	0.040		0.037	0.037	

N represents the total number of replicates within treatments. Region abbreviations represent: AR = Argentina, CA = California, CH = Chile, EU = Eurasia. Asterisks indicate significant overall treatment effects (* = p<0.05, ** = p<0.01, *** = p<0.001, **** = p<0.0001). Superscripts indicate significant differences after post-hoc Tukey HSD contrasts.

#### Root Biomass

**Figure 2:**
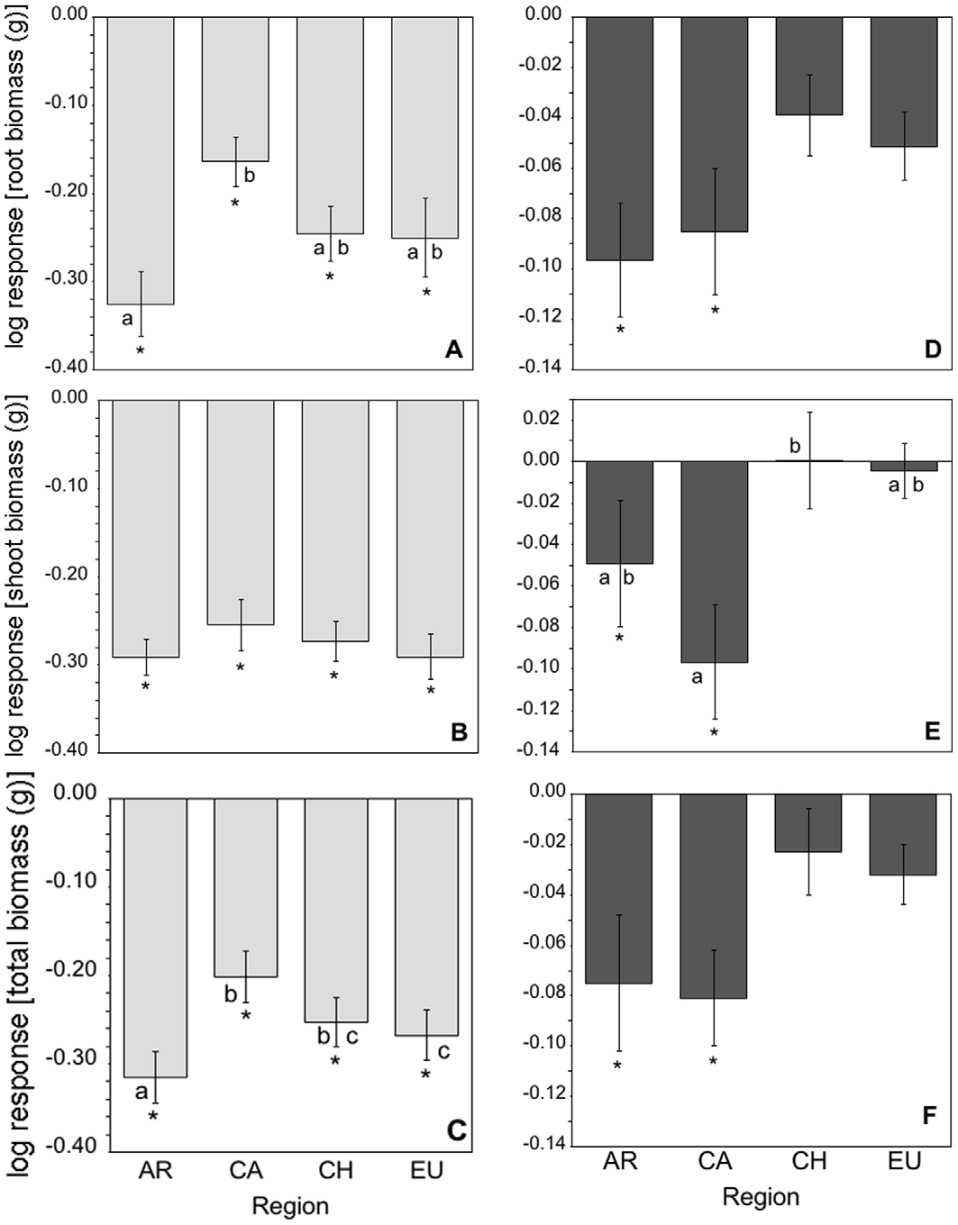
Log response ratios illustrating the net effects of soil microbes (A–C) and soil training (D–F) on *Centaurea* root biomass (top row; A, D), shoot biomass (middle row; B, E), and total biomass (bottom row; C, F) by source regions. See Methods for equations used to calculate log response ratios. Negative values indicate negative effects of soil microbes (A–C) and negative plant-soil feedbacks (D–F). Region abbreviations are as in Table 2. Bars represent means ±1. S.E.M. Asterisks represent significant differences in corresponding response variables when grown in field vs. sterilized soils (A–C), or soils trained by *Centaurea* vs. grasses (D–F) after t-test analysis. Different letters represent significant differences among regions after Tukey HSD post-hoc tests.

Root biomass did not differ across regions (Table 2). Source population nested within region accounted for 22.5% of the total variance in root biomass. Soil microbes and soil training by conspecifics reduced root biomass in all regions (Table 2; Figure 2A, D). However there was also a region*sterilization interaction (*F*_3,20_ = 7.68, *p* = 0.04) with the most negative effects of soil microbes on root biomass in Argentinean soils and the least negative impacts in California soils (Figure 2A). *Centaurea* in Argentinean and Californian soils generated negative feedbacks (t-test *p* = 0.007 and 0.001, respectively), whereas Chilean and Eurasian *Centaurea* showed no difference in root biomass between soil training treatments.

#### Shoot Biomass

Shoot mass was highest for Eurasian plants, intermediate for Argentinean and Californian plants, and lowest for Chilean plants (Table 2). Soil microbes and training by conspecifics significantly reduced shoot biomass (Table 2). Source population nested within region accounted for 34.7% of the total variance in shoot biomass. As for root biomass, there was also a significant region*training interaction (*F*_3,20_ = 3.13, *p* = 0.049) where plants generated negative feedbacks only in soils from Argentina and California (t-test *p* = 0.047 and 0.0003, respectively; Figure 2E). There was no variation in the effects of soil microbes on shoot mass among regions (Figure 2B).

#### Total Biomass

There was no significant difference in total biomass among plants grown in soils from different regions (Table 2). Eurasian *Centaurea* were the largest and grew 18% larger than Chilean *Centaurea.* Population nested within Region accounted for 30.5% of the total variance in total biomass.

Soil microbes significantly reduced total biomass of *Centaurea* in field soils from all regions (Table 2). However, there was a marginally significant region*sterilization interaction (*F*_3,20_ = 2.62, *p* = 0.08) where the greatest negative effects of microbes were in Eurasian and Argentinean soils with weaker effects in Chilean and California soils (Figure 2C; Tukey HSD at **α** = 0.05).

*Centaurea* grown in soil trained by conspecifics were significantly smaller than in grass-trained soil (Table 2). This pattern was driven mainly by Argentina and California soil treatments, illustrated by a significant interaction between soil training and soil origin (*F*_3,20_ = 3.31, *p* = 0.04; Figure 2F). Consistent with the pattern of shoot biomass and the trend in root biomass, *Centaurea* generated significant negative feedbacks, but only in Argentinean (t-test *p* = 0.012) and Californian (t-test *p* = 0.0002) soils. When we conducted the analysis by grouping populations demographically as either “rapidly spreading” or “stable or slowly spreading,” a clear and strong pattern emerged: *Centaurea* from populations that are spreading rapidly (Argentina and California) generated negative feedbacks that differed significantly from the neutral feedbacks generated by *Centaurea* from populations that are relatively stable (Chile and Eurasia; Figure 2D-F).

#### Root:Shoot Ratio

**Figure 3:**
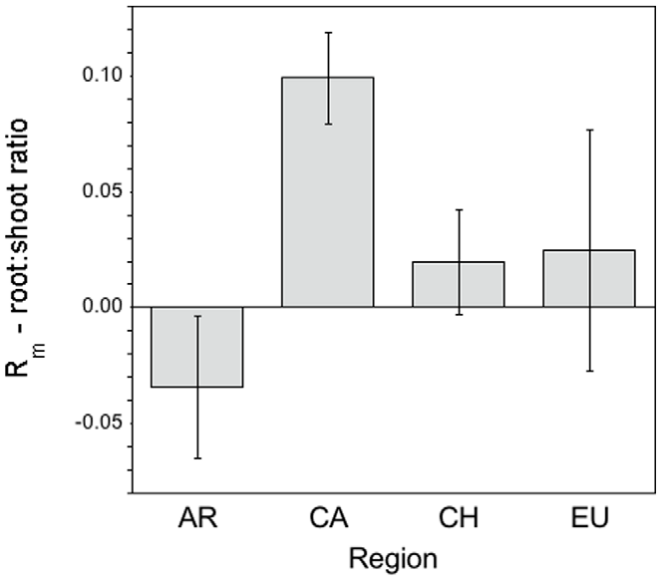
Log response ratios illustrating the net effects of soil microbes on root:shoot ratio of *Centaurea* from different regions. Negative bars represent declines in root:shoot ratios in response to soil microbes, while positive bars represent increases in root:shoot ratio in response to soil microbes. Region abbreviations are as in Table 2. Bars represent means ±1. S.E.M. Asterisks represent significant differences in root:shoot ratio when grown in field vs. sterilized soils after t-test analysis. Different letters represent significant differences among regions after Tukey HSD post-hoc tests.

*Centaurea* grown in Chilean soils had significantly greater RSR than in Eurasian and Californian soils (Table 2; Tukey HSD at **α** = 0.05). Sterilization significantly reduced RSR across treatments (Table 2), but this was driven mainly by plants in California soils (Figure 3), while sterilization tended to increase RSR of plants in Argentinean soils (region*sterilization *F*_3,20_ = 2.73, *p* = 0.07; Tukey HSD at **α** = 0.05). Plants in soils trained by grasses had a higher RSR (Table 2) in all regions except California, where there was no effect of soil training.

### Common Garden Experiment

*Centaurea* grown from seeds collected from all four regions did not differ in root biomass (*F*_3,36_ = 1.00, *p* = 0.40), shoot biomass (*F*_3,36_ = 1.81, *p* = 0.16), total biomass (*F*_3,36_ = 1.93, *p* = 0.14), or RSR (*F*_3,36_ = 0.07, *p* = 0.9) when grown in the same soil environment, which is consistent with past studies [24]. However, Argentinean plants took more than twice as many days to germinate than plants from any other region (*F*_3,36_ = 21.41, *p*<0.0001; Tukey HSD at **α** = 0.05). Since all plants were grown for the same number of days after germination (see Methods), the delayed germination of Argentinean *Centaurea* did not contribute to differences in biomass.

### Plant soil feedbacks and *Centaurea* demography

**Figure 4:**
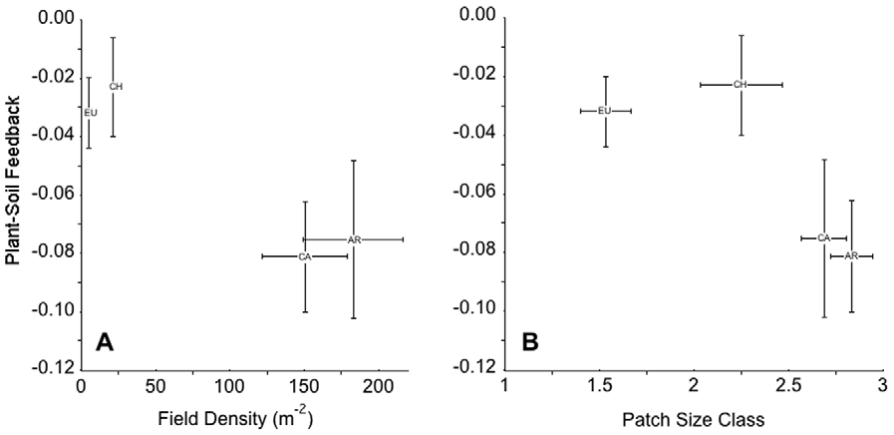
Plant soil feedback responses (R_FB_) of *Centaurea* total biomass (g) from four regions as a function of (A) local field density and (B) patch size class from each region. All data represent means ±1 S.E.M. Region abbreviations are as in Table 2. Note: the x-error bars for EU and CH in (A) were smaller than the marker labels.

Field densities and patch sizes of *Centaurea* in expanding populations of Argentina and California were much higher than populations in Chile and native Eurasia, where populations are smaller and relatively stable (Figure 4). However, *Centaurea* generated the strongest negative plant-soil feedbacks in regions where it was the most invasive (Figure 4).

## Discussion

### Is *Centaurea* released from natural enemy pressure from soil microbes in introduced regions?

Biogeographical variation in pathogen pressure has been well documented [13], [14], [19], [20], [23], [25], [36] and is one basis for the Enemy Release Hypothesis for species invasions [11]–[13]. In our study, soil microbes reduced *Centaurea* performance from all regions, but to varying degrees. Argentinean plants were most inhibited by their local soil microbes, while California plants were least affected. The effects of soil microbes on Eurasian plants, which have presumably coevolved relationships, were intermediate. Thus, Argentinean plants which experienced the heaviest enemy pressure from soil microbes may compensate by allocating less to root tissues, while California plants that experienced the least negative impacts from soil microbes actually invested the most in root tissue, a pattern that may emerge if plants were experiencing heavy pressure from soil-borne pathogens [37]. On the other hand, changes in RSR may also reflect direct losses of root tissue due to pathogen infection. A recent study examining effects of soil feedbacks on another invasive aster, *Chromolaena odorata*, also reported a lack of enemy release in soils in the introduced range, but demonstrated increases in allocation to above-ground tissues [38]. Patterns such as these illustrate how plasticity of resource allocation may be associated with the success of invasive species.

Our results suggest that while *Centaurea* may escape from native Eurasian natural enemy pressure in some introduced populations (in California), they may experience even more pathogen pressure in other introduced regions (such as Argentina). Although we did not identify specific microbes in our soils, the goals of our experiments were to examine the broad patterns of soil microbe effects throughout *Centaurea'*s distribution, rather than the specific microbes responsible for these effects. However, other analyses have shown that soils from *Centaurea*'s native range contain higher densities of at least one fungal pathogen, *Pythium* sp., than soils from introduced populations (*K. Reinhart* personal communication). This pattern illustrates that mechanisms such as enemy release may be causal factors in the spread of an introduced species in some parts of its range, but may not determine invasive success in all non-native regions.

### How do plant-soil feedbacks affect variation in invasive success throughout native and introduced regions?

Many studies of plant invasions and soil microbes have shown that invasive plants often generate weaker negative or even positive plant-soil feedbacks in introduced regions, potentially releasing invasive plants from one component of density-dependent regulation [18], [20], [22], [23], [46]. Our results show the opposite pattern for *Centaurea solstitialis*, which generated significant *negative* feedbacks, but only in soils where populations are spreading most rapidly (Argentina and California). In addition, germination of Argentinean plants was strongly and negatively affected while Eurasian plants had positive germination responses to soils trained by conspecifics. One interpretation is that plant-soil feedbacks do not affect invasion success of *Centaurea* in California and Argentina. Perhaps other mechanisms drive *Centaurea*'s invasion in these regions and allow the weed to overcome the effects of negative plant-soil feedbacks. Another possible interpretation of our results is that negative feedbacks enhance outward spread since plants perform relatively better in adjacent uncolonized areas than in established stands, while empty niches, enemy escape, or other unknown factors that may lead to rapid growth rates increase local scale dominance and persistence. Other researchers have found similar results in a recent plant-soil feedback experiment of the highly invasive tree *Sapium sebiferum* [39]. Of the five species examined, *Sapium* was the only one to generate negative feedbacks in its introduced range, despite being the only invasive species; although that study lacked biogeographical comparisons, it demonstrated that a highly invasive species can perform unusually well even while experiencing strong negative feedbacks. However, the authors [39] suggested that these negative frequency dependant forces may limit *Sapium*'s long-term persistence. The negative feedbacks we observed in *Centaurea* may also limit its long-term persistence, as has been observed in some Argentinean populations [25]. However, *Centaurea* continues its rapid spread in California, including high-elevation alpine habitats [40]. Although negative feedbacks often reduce species' dominance and enhance species coexistence [17], [18], *Centaurea* manages to invade despite negative feedbacks. Here we propose that these feedbacks may also contribute to the *spread* of invaders, by promoting *Centaurea* growth into uninvaded regions.

Even though *Centaurea* generates negative feedbacks where it is spreading most rapidly, its effects on the soil community may be even more detrimental to native plant species, thus creating an environment where *Centaurea* can dominate despite experiencing negative feedbacks. For example, *Centaurea* may accumulate generalist pathogens in the rhizosphere that negatively affect conspecifics, but with even greater negative effects on its competitors [17], [41], thereby cultivating a soil community that gives a net benefit to *Centaurea* through handicapping competing species. This mechanism has been termed the ‘accumulation of local pathogens’ [42] and has been supported by studies of other invasive weeds that also excel in the absence of enemy release, including *Ammophila arenaria* and *Chomolaena odorata* [43], [44]. Because we did not measure the effects of soil training by *Centaurea* on other plant species in the community, these mechanisms remain speculative.

A recent study demonstrated negative associations between the degree of enemy release and the spread of alien plants in Europe [45], also contrary to the paradigm of enemy release in invasion biology. Although this study did not examine soil microbes it reflects the potential trade-offs between plant spread and enemy attack, such that rapidly spreading plants accumulate more pathogens in regions where they are spreading. These trade-offs may also be operating on the variable success of introduced *Centaurea* populations and could also explain patterns observed in this study.

#### Regional variation and evolution of invasiveness

Invasive species are often larger and more vigorous in their introduced than native ranges [47]–[49], but our results did not unequivocally support this pattern. Although Argentinean and Californian *Centaurea* populations are among the most highly invasive *Centaurea* populations in the world, the biomass of *Centaurea* from these regions did not differ from native Eurasian *Centaurea*, while *Centaurea* from slowly spreading introduced Chilean populations were the smallest. These data are consistent with previous common garden studies that found no differences in biomass among *Centaurea* from different regions [25], except for delayed germination in Argentinean *Centaurea* [50]. Since plant size did not differ in the common garden study and used the seeds from the same populations as in the Feedback Experiment, the differences in biomass reported in the latter study are likely due to treatment effects rather than maternal effects or genetic differences in growth in plants from different regions. Thus, the low biomass of Chilean plants in the soil feedback experiment is likely due to soil microbes. Chilean soil microbes may inhibit *Centaurea*, which could explain why these populations do not spread as rapidly as in the other introduced regions. Further, the similarities of biomass among plants in the common garden experiment provide some preliminary evidence that *Centaurea* has not evolved to be larger in non-native ranges. While we lacked population-level replication in our common garden experiment, a second, well replicated experiment also found no differences in biomass among populations from native and non-native regions [51]. Finally, while variation in invasion success among introduced regions may reflect time since introduction, *Centaurea* was introduced into all the non-native regions examined in this study within a 50-year span [32], so this is unlikely. The success and spread of *Centaurea* may be influenced more by contemporary ecological interactions, such as those with soil biota, than by evolutionary shifts in allocation and competitiveness.

#### Conclusions

In total, the most invasive and rapidly spreading *Centaurea* populations from Argentina and California were those that generated negative plant-soil feedbacks. Interestingly, soil microbes had the most negative net effects on Argentinean plants and least negative effects on California plants. Thus, negative feedbacks may influence the spread of a plant invader despite differences in potential direct effects of soil microbes. These results highlight the importance of examining geographic variation in species interactions and demonstrate the variability in mechanisms driving invasions on broad geographical scales.

This study is among the first to take a broad, global biogeographic scope across different non-native ranges that span a gradient of invasiveness. Through this approach, we found substantial variation in the interaction of *Centaurea* with soil microbes, suggesting that different mechanisms may influence its invasive success in different introduced regions. It is likely that such biogeographic variation in species interactions and mechanisms controlling invasive spread are the norm rather than the exception [36], [52]. Although this introduces challenges in determining causal factors or predicting invasion patterns, there seems to be a biogeographical mosaic of species interactions that contribute to variation in invasion success, highlighting the ecological and biogeographical complexity of biological invasions.

## Supporting Information

Table S1 Location and elevation of populations used for seed and soil collections for greenhouse experiments. *Centaurea solstitialis* seeds are abbreviated as Cs.
(XLS)
